# Supramalleolar osteotomy with medial distraction arthroplasty for ankle osteoarthritis with talar tilt

**DOI:** 10.1186/s13018-019-1168-z

**Published:** 2019-05-06

**Authors:** Hong-Mou Zhao, Xiao-Dong Wen, Yan Zhang, Jing-Qi Liang, Pei-Long Liu, Yi Li, Jun Lu, Xiao-Jun Liang

**Affiliations:** Foot and Ankle Surgery Department, Honghui Hospital of Xi’an Jiaotong University, Xi’an, 710054 China

## Abstract

**Background:**

An increased preoperative talar tilt (TT) angle was reported to be positively correlated with treatment failure after supramalleolar osteotomy (SMOT) for varus ankle osteoarthritis. Distraction arthroplasty was reported to have the ability to correct increased TT angles. The purpose of the current study was to compare the outcomes between SMOT with and without medial distraction arthroplasty (MDA) in the treatment of varus ankle osteoarthritis with increased TT angles.

**Methods:**

We retrospectively reviewed the functional outcomes and radiological findings of 34 patients who underwent SMOT with or without MDA for varus ankle osteoarthritis with increased TT angles. The American Orthopaedic Foot and Ankle Society (AOFAS) ankle-hindfoot score and Ankle Osteoarthritis Scale (AOS) scores were used for functional evaluation. The tibial anterior surface (TAS) angle, talar tilt (TT) angle, tibial medial malleolar (TMM) angle, talocrural (TC) angle, tibial lateral surface (TLS) angle, and hindfoot alignment (HFA) angle were evaluated preoperatively and at the time of the last follow-up.

**Results:**

In the SMOT group, the AOFAS score and AOS pain and function scores were significantly improved (*P* < 0.01 for each) at a mean follow-up of 61 months. The TAS, TT, TC, TLS, and HFA angles were all significantly improved (*P* < 0.01 for each). Similarly, in the SMOT with MDA group, the AOFAS score, AOS pain and function scores, and the TAS, TT, TC, TLS, and HFA angles were all significantly improved postoperatively (*P* < 0.01 for each). When comparing the two groups, the postoperative TT angle was significantly smaller in the SMOT with MDA group (*P* = 0.023) than in the SMOT group. In addition, the failure rate of TT angle correction was significantly higher in the SMOT group (*P* = 0.016) than in the SMOT with MDA group.

**Conclusion:**

SMOT is a promising procedure for functional improvement and malalignment correction for varus ankle osteoarthritis, even in patients with increased talar tilt. If SMOT is combined with MDA, there can be an improvement in the correction of the increased talar tilt.

**Level of evidence:**

Level III, a retrospective comparative study

## Background

Ankle osteoarthritis is a progressive disease characterized by the degeneration of articular cartilage and often develops asymmetrically with a concomitant varus deformity [1, 2]. The uneven pressure on the articular surface has a close relationship with the degeneration of cartilage, which may induce osteoarthritic changes and the progression and development of the disease [3]. Supramalleolar osteotomy (SMOT) is based on the theory of joint pressure redistribution during weight-bearing actions and aims to delay the progression and development of osteoarthritis [4, 5]. Clinical and biomechanical studies reported that SMOT could realign the weight-bearing line, restore the congruence of the tibiotalar joint [6–10], decrease the contact pressure of the medial part of the tibiotalar joint [11, 12], and even reverse the radiological ankle osteoarthritis stages [5, 13, 14].

However, the role of the talar tilt (TT) angle on SMOT is controversial. Some studies reported that increased preoperative TT angles were correlated with increased postoperative TT angles [5, 14–16], and large postoperative TT angles were positively correlated with treatment failure [14, 16]. Although some authors reported that no radiological outcomes seemed to have a significant influence on the clinical outcomes [17], many doctors and patients are still worried about the potential for poor outcomes.

Ankle joint distraction arthroplasty was first reported in 1995 by van Valburg and his colleagues [18] and has evolved as an option for cartilage preservation in patients with osteoarthritis [19, 20]. Although distraction arthroplasty cannot correct the bony deformities, it may shift the force from the hindfoot to the valgus at the joint level and give the medial structure a distraction. We found that in our distraction patients, the increased TT angles were corrected and maintained during follow-up [21]. Therefore, we evaluated the use of medial distraction arthroplasty (MDA) in our SMOT patients to correct their increased TT angles, and we observed positive outcomes [18]. Thus, we hypothesize that distraction arthroplasty may play a role in realignment surgery and may help restore the congruence of the ankle joint. The purpose of the current study was to retrospectively analyze and compare the clinical and radiological outcomes of SMOT with or without MDA for the treatment of varus ankle osteoarthritis with large preoperative TT angles.

## Methods

The current study was approved by the research board of our hospital. The authors retrospectively studied the outcomes of SMOT with or without MDA in the treatment of varus ankle osteoarthritis with increased TT angles between January 2010 and October 2016. The inclusion criteria were as follows: (1) adults who were at least 18 years of age; (2) patients with a tibial articular surface (TAS) angle of less than 84°; (3) patients with varus ankle osteoarthritis; (4) patients with clinical symptoms, such as pain with walking and limitation of daily and recreational activities; (5) patients with a TT angle larger than 5° [15]; (6) patients who were treated with SMOT with or without MDA; and (7) patients with at least 2 years of follow-up. The exclusion criteria were as follows: (1) patients with neurological disorders, (2) patients with rheumatoid arthritis, (3) patients with Charcot arthropathy, (4) patients with Charcot-Marie-Tooth deformity, (5) patients with acute or chronic infections of the ankle joint, and (6) patients who required reoperation after SMOT failure.

Finally, 16 cases in the SMOT group and 18 cases in the SMOT with MDA group were included in the study. There were 11 males and 23 females, and the mean age was 54.8 (range, 23–77) years. According to the modified Takakura ankle osteoarthritis stage, there were 13 patients with stage 3a osteoarthritis, 19 patients with stage 3b osteoarthritis, and 2 patients with stage 4. The basic information of the included patients is listed in Table 1. Between the 2 groups, there were more cases of autografts in the SMOT group and more allografts in the SMOT with MDA group (*P* = 0.014). The mean follow-up time in the SMOT group was longer than in the SMOT with MDA group (*P* < 0.001). There were no significant differences among the other information with the available numbers.

**Table 1:** Basic information and preoperative parameters of the two groups

	SMOT (*n* = 16)	SMOT with MDA (*n* = 18)	*P* value
Male/female	5/11	6/12	0.897
Age, year	53.4 ± 10.2	56.2 ± 11.9	0.470
Left/right	6/10	5/13	0.717
Fibular osteotomy	8	7	0.515
Brostrom procedure	3	5	0.693
Calcaneal osteotomy	1	2	0.998
Takakura stage 3a/3b/4	7/8/1	6/11/1	0.805
Auto-/allograft	11/5	4/14	0.014
Follow-up, month	61.4 ± 20.6	35.6 ± 15.3	< 0.001
Preoperative outcomes			
AOFAS score, point	47.3 ± 14.9	49.2 ± 12.0	0.683
AOS pain, point	5.6 ± 0.9	5.9 ± 0.8	0.311
AOS function, point	5.9 ± 1.1	6.2 ± 0.9	0.389
ROM of ankle, degree	34.7 ± 7.8	35.7 ± 7.2	0.700
Preoperative radiological parameters (degree)			
TAS	80.8 ± 3.0	81.6 ± 2.2	0.378
TT	11.2 ± 3.4	12.3 ± 4.0	0.397
TMM	32.6 ± 7.3	34.9 ± 8.6	0.410
TC	71.2 ± 2.9	69.5 ± 4.1	0.177
TLS	76.8 ± 3.6	75.4 ± 3.4	0.252
HFA*	16.7 ± 4.4	17.4 ± 5.3	0.755

*SMOT* supramalleolar osteotomy, *MDA* medial distraction arthroplasty, *AOFAS* American Orthopaedic Foot and Ankle Society ankle-hindfoot score, *AOS* the Ankle Osteoarthritis Scale, *ROM* range of motion, *TAS* tibial articular surface angle, *TT* talar tilt angle, *TMM* tibial medial malleolar angle, *TC* tibiocrural angle, *TLS* tibial lateral surface angle, *HFA* hindfoot alignment angle

*The case number in SMOT group was 8, and in SMOT with MDA group was 14

### Operative technique

All of the included patients were treated with medial opening wedge SMOT. The surgical technique of SMOT for fibular osteotomy has been well described in a previous study [13]. The tibial osteotomy was approximately 5 cm proximal to the medial malleolar tip. Before osteotomy, a K-wire was placed from the medial malleolus to the lateral cortex to guide the osteotomy. The osteotomy plane slightly inclined from the medial-superior to the lateral-inferior and ended at the syndesmosis level. Subsequently, the osteotomy was performed with the use of a wide saw blade, and the lateral cortex was carefully preserved. According to the preoperative plan, the aim for the TAS angle was 90 to 92°, and the aim for the tibial lateral surface (TLS) angle was 80 to 85°. The patient chose whether an iliac autograft or allograft was used to fill the tibial osteotomy site. The resulting sharp spike of the medial distal tibia bone from the opening wedge was removed, if necessary. Then, the osteotomy site was internally fixed with the use of a medial plate.

If the talocrural (TC) angle decreased by more than 5° in comparison with the uninjured site [22], the fibula had a rotational deformity, or there was interference with the reduction of the tibial plafond and talus, then a fibular osteotomy was performed with a lateral approach at the same level or higher than that for the tibial osteotomy. The fibular osteotomy sites were internally fixed with plates.

If the patient had chronic ankle joint instability or was unstable after lateral osteophyte debridement, a modified Brostrom procedure was used to ensure the lateral stability of the ankle joint. If the patient still had varus deformity of the hindfoot after SMOT, a calcaneal osteotomy was used to further improve the lateral movement of the weight-bearing site in the hindfoot.

In the SMOT with MDA group, after the SMOT incisions were closed, an external fixator was applied. One pin was placed into the calcaneus under fluoroscopy, one half pin was placed into the talus from the medial side to the lateral side, and one or two additional pins were placed into the tibia. A fixed external fixator, which allowed further distraction without permitting ankle joint motion, was applied. With the distraction of the talus pin during the operation, the TT angle was corrected to 0° or negative degrees and was verified fluoroscopically.

The postoperative rehabilitation protocol included active and passive motion exercises of the ankle and midfoot and forefoot joints, isotonic and isometric exercises of the leg, and the use of a night splint beginning on the second postoperative day in the SMOT group. Patients were permitted to be partially weight-bearing 6 weeks postoperation. Full weight-bearing activities began after the osteotomy site achieved bony union that was confirmed radiographically. The external fixator was used for 10 to 12 weeks in the SMOT with MDA group. The postoperative rehabilitation protocol in the SMOT with MDA group was similar to that of the SMOT group, except that weight-bearing actions and motions of the ankle joint were prohibited. After the external fixator was removed, the patient was permitted to begin partial weight-bearing activities for 1 month and then full weight-bearing activities after the osteotomy site achieved bony union, which was confirmed radiographically.

### Assessments

**Fig. 1:**
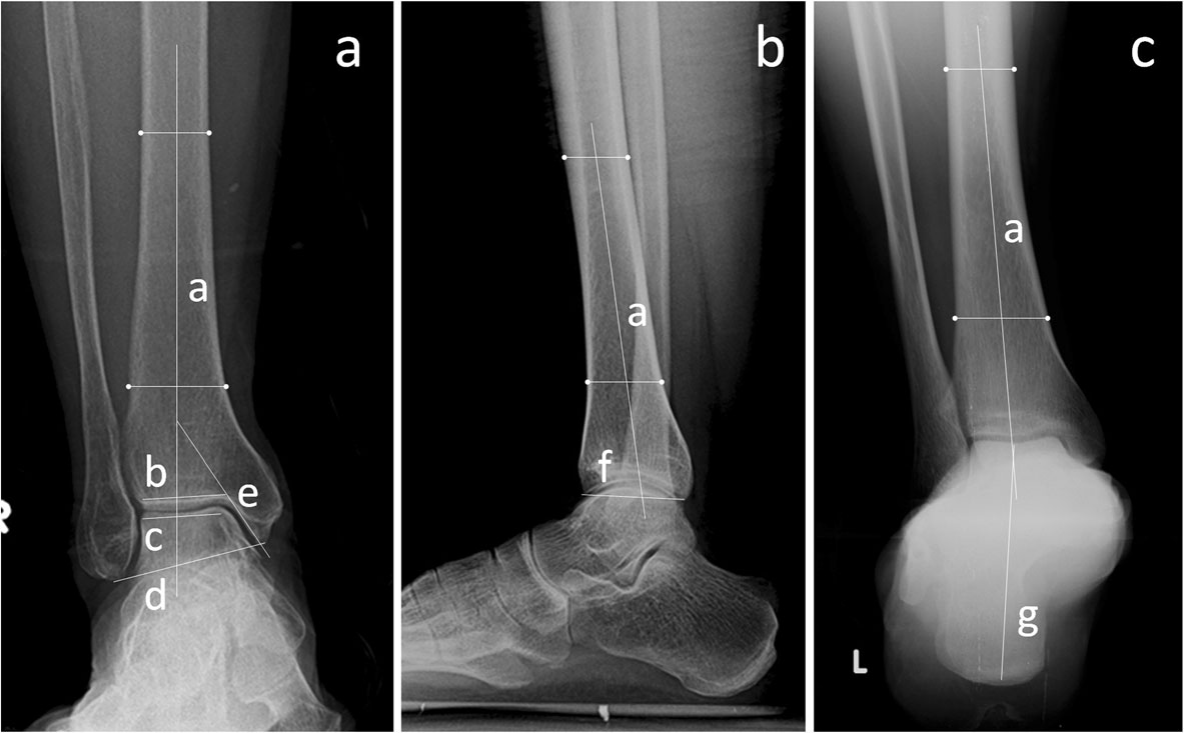
Anterior-posterior view of the ankle (**a**). Tibial articular surface (TAS) angle, the angle between line a and b; talar tilt (TT) angle, the angle between line b and c; talocrural (TC) angle, the angle between line a and d; and tibial medial malleolar (TMM) angle, the angle between line a and e. Lateral view of the ankle (**b**). The tibial lateral surface (TLS) angle, the angle between tibial axis line a and articular tangent line f. Saltzman view of the ankle and the hindfoot alignment (HFA) angle, the line between a and g (**c**)

The radiological evaluations visualized the TAS, TT, TC, and tibial medial malleolar (TMM) angles in an anterior-posterior ankle view, the TLS angle in a lateral ankle view, and the hindfoot alignment (HFA) angle in the Saltzman view (Fig. 1). All of the included measurements on the weight-bearing radiographs were performed by two observers independently.

The American Orthopaedic Foot and Ankle Society (AOFAS) ankle-hindfoot score and the Ankle Osteoarthritis Scale (AOS) were used to evaluate the functional outcomes preoperatively and postoperatively [23, 24]. Treatment failure was defined as the patient requiring reoperation because of the relative reasons for the initial operation. The reoperations included osteotomy, arthrodesis, and arthroplasty; patients with no symptoms after hardware removal were not included. The functional outcomes and radiological parameters before failure relative to those after reoperation were included as the patients’ final results.

To analyze the changes in the radiographical grade, stages 1, 2, 3a, 3b, and 4 of the modified Takakura classification system were assigned quantitative scores of 1, 2, 3, 4, and 5, respectively.

### Statistical analyses

Descriptive statistics were calculated as the means ± standard deviation. Statistical analyses of the included data were performed using Student’s *t* test, Pearson’s chi-square test, or Fisher’s exact test with the level of significance set at *α* = 0.05. The statistical analyses were performed with SPSS 17.0 software (SPSS, Inc., Chicago, IL).

## Results

### Functional and radiological improvement in the two groups

All patients in both groups achieved bony unions without incision-related complications. No patient in either group had other soft tissue complications. Pin tract infections of the tibia occurred in two patients in the SMOT with MDA group. Both of these patients were treated with dressing changes and oral antibiotics; no patient needed early removal of the external fixator. When compared with the preoperative conditions, both groups achieved significant improvement in the AOFAS scores (*P* < 0.001, Table 2), as well as in the AOS pain and functional scores (*P* < 0.01). However, the range of motion (ROM) of the ankle joint was not significantly improved with the available numbers. The modified Takakura stages in both groups decreased significantly (*P* < 0.001). In both groups, all of the radiological parameters, including the TAS, TT, TMM, TC, TLS, and HFA angles, improved significantly (*P* < 0.05).

**Table 2:** Comparison of the preoperative and last follow-up time functional outcomes and radiological parameters

	SMOT (*n* = 16)		*P* value	SMOT with MDA (*n* = 18)		*P* value
	Preoperation	Last follow-up		Preoperation	Last follow-up	
Functional outcomes						
AOFAS, point	47.3 ± 14.9	77.4 ± 19.9	< 0.001	49.2 ± 12.0	84.5 ± 6.7	< 0.001
AOS pain, point	5.6 ± 0.9	3.5 ± 2.3	0.002	5.9 ± 0.8	2.4 ± 0.6	< 0.001
AOS function, point	5.9 ± 1.1	3.6 ± 2.0	< 0.001	6.2 ± 0.9	2.9 ± 0.9	< 0.001
ROM of ankle, degree	34.7 ± 7.8	36.4 ± 7.6	0.537	35.7 ± 7.2	37.8 ± 6.3	0.358
Takakura stage	3.6 ± 0.6	1.9 ± 1.0	< 0.001	3.8 ± 0.6	1.4 ± 0.6	< 0.001
Radiological parameters						
TAS	80.8 ± 3.0	89.1 ± 2.0	< 0.001	81.6 ± 2.2	89.8 ± 1.6	< 0.001
TT	11.2 ± 3.4	2.9 ± 1.7	< 0.001	12.3 ± 4.0	1.8 ± 0.9	< 0.001
TMM	32.6 ± 7.3	27.7 ± 4.6	0.031	34.9 ± 8.6	28.5 ± 6.2	0.015
TC	71.2 ± 2.9	77.9 ± 2.8	< 0.001	69.5 ± 4.1	76.9 ± 3.7	< 0.001
TLS	76.8 ± 3.6	80.5 ± 2.3	0.002	75.4 ± 3.4	79.5 ± 3.0	0.001
HFA*	16.7 ± 4.4	4.2 ± 2.6	< 0.001	17.4 ± 5.3	3.1 ± 1.8	< 0.001

*SMOT* supramalleolar osteotomy, *MDA* medial distraction arthroplasty, *AOFAS* American Orthopaedic Foot and Ankle Society ankle-hindfoot score, *AOS* the Ankle Osteoarthritis Scale, *ROM* range of motion, *TAS* tibial articular surface angle, *TT* talar tilt angle, *TMM* tibial medial malleolar angle, *TC* tibiocrural angle, *TLS* tibial lateral surface angle, *HFA* hindfoot alignment angle

*The case number in SMOT group was 8, and in SMOT with MDA group was 14

### Functional and radiological comparison between the two groups

When comparing the postoperative functional outcomes of the two groups, the AOFAS scores and AOS pain and functional scores were not significantly different at the time of the final follow-up with the available numbers (Table 3). Three patients in the SMOT group underwent ankle arthrodesis at 17, 26, and 61 months postoperatively because of pain and dysfunction. No patients in the SMOT with MDA group required arthrodesis by the end of the follow-up time. However, the two groups demonstrated no significant differences in failure rate with the available numbers. Additionally, the postoperative modified Takakura stages were not significantly different with the available numbers.

**Table 3:** Functional outcomes and radiological parameters between the two groups at the last follow-up time

	SMOT (*n* = 16)	SMOT with MDA (*n* = 18)	*P* value
Functional outcomes			
AOFAS, point	77.4 ± 19.9	84.5 ± 6.7	0.163
AOS pain, point	3.5 ± 2.3	2.4 ± 0.6	0.059
AOS function, point	3.6 ± 2.0	2.9 ± 0.9	0.189
ROM of ankle, degree	36.4 ± 7.6	37.8 ± 6.3	0.561
Failure rate	18.8% (3 / 16)	0	0.094
Takakura stage	1.9 ± 1.0	1.4 ± 0.6	0.083
Radiological parameters			
TAS	89.1 ± 2.0	89.8 ± 1.6	0.266
TT	2.9 ± 1.7	1.8 ± 0.9	0.023
TMM	27.7 ± 4.6	28.5 ± 6.2	0.675
TC	77.9 ± 2.8	76.9 ± 3.7	0.386
TLS	80.5 ± 2.3	79.5 ± 3.0	0.288
HFA*	4.2 ± 2.6	3.1 ± 1.8	0.254
TT not corrected	31.3% (5/16)	0	0.016

*SMOT* supramalleolar osteotomy, *MDA* medial distraction arthroplasty, *AOFAS* American Orthopaedic Foot and Ankle Society ankle-hindfoot score, *AOS* the Ankle Osteoarthritis Scale, *ROM* range of motion, *TAS* tibial articular surface angle, *TT* talar tilt angle, *TMM* tibial medial malleolar angle, *TC* tibiocrural angle, *TLS* tibial lateral surface angle, HFA hindfoot alignment angle

*The case number in SMOT group was 8, and in SMOT with MDA group was 14

**Fig. 2:**
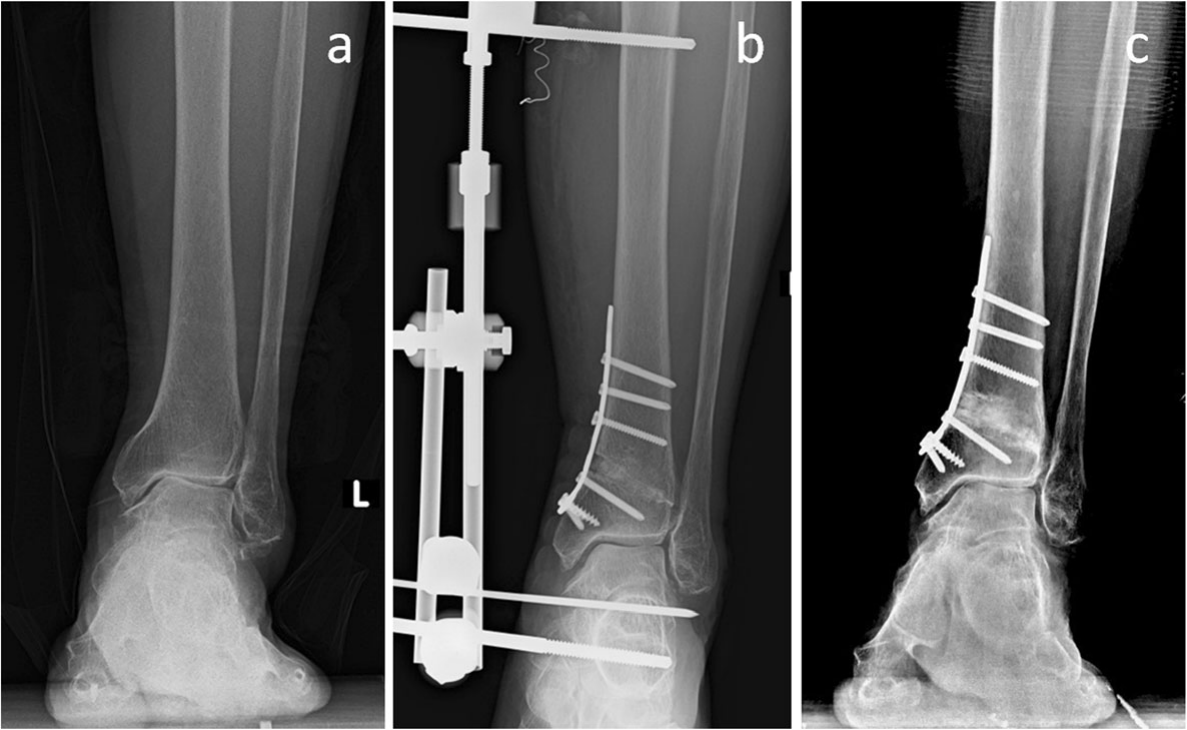
A 62-year-old female patient. The preoperative anterior-posterior view showed stage 3b varus ankle osteoarthritis (**a**). The patient was treated with supramalleolar osteotomy and medial distraction arthroplasty (**b**). The 1-year postoperative scan showed normal alignment of the ankle joint; the talar tilt angle was decreased to 1.7°, and the modified Takakura stage was improved to stage 1 (**c**)

When comparing the postoperative radiological parameters of the two groups, the TT angle in the SMOT with MDA group was significantly smaller than that of the SMOT group (*P* = 0.023). All of the TT angles in the SMOT with MDA group were corrected to normal angles (≤ 4° [15], Fig. 2). However, the TT angles of five patients in the SMOT group were not corrected to normal angles (*P* = 0.016). Among these five patients, two patients had fused joints, two were still symptomatic and treated conservatively, and only one reported a good outcome. The other postoperative radiological parameters, including TAS, TMM, TC, TLS, and HFA angles, were not significantly different with the numbers available.

## Discussion

Osteoarthritis is a slowly progressive degenerative joint disorder that, in most cases, is diagnosed at a late stage after the onset of accompanying clinical symptoms. Ankle joint osteoarthritis is one of the most common joint diseases and is a significant source of pain and disability for middle-aged and elderly people throughout the world [25]. Joint-sacrificing procedures, including total ankle replacement and arthrodesis, are used for painful end-stage ankle osteoarthritis. However, both procedures have disadvantages and are associated with limited long-term benefits [1, 26, 27]. Realignment osteotomy, which is based on the theory that uneven pressure on the articular surface of the lower extremities may induce arthritis [3], is used to redistribute the weight-bearing pressure on the joint to delay the progression and development of osteoarthritis. The midterm results of SMOT showed good outcomes for pain relief, functional improvement, and a return to sports and recreational activities [5–10, 14, 16, 17, 22, 28–30]. However, the treatment is still controversial, especially in patients with increased preoperative TT angles.

Talar tilt is common in varus ankle osteoarthritis and is not a bony deformity that results in the incongruence of the tibiotalar joint. The initial reason for the increased TT angle may be due to a lateral collateral ligament injury of the ankle joint. However, this will result in a medial shift of the center of the talus and joint loading axis [12] and will increase the tension and expansion of the lateral soft tissue to further increase the deformity. Some authors reported that SMOT could significantly decrease the TT angle [7, 9, 17, 22, 31]; however, some did not observe the same decrease [6, 14–16]. Tanaka et al. [14] reported that all patients with a preoperative TT angle larger than 10° in the joint space did not improve to a normal angle. Lee et al. [16] reported that the preoperative TT angle was correlated with the postoperative TT angle and recommended that the optimal threshold for predicting a large postoperative TT angle was a preoperative TT angle of 7.3°. Ahn et al. [6] reported that the TT angle was not significantly corrected after SMOT. In our SMOT cases, the TT angles of five (31.3%) cases did not correct to a normal angle postoperatively, and two of the five (40%) patients experienced treatment failure. Therefore, a challenge for surgeons is effectively correcting the increased TT angle to a normal angle in varus ankle osteoarthritis patients.

Joint distraction arthroplasty in the treatment of severe ankle osteoarthritis shows significant and prolonged improvement in pain and functional ability in open prospective studies as well as in a randomized controlled trial [32, 33]. Our previous study reported that distraction arthroplasty using talus medial half pins could shift the force from the hindfoot to the valgus, thereby distracting the medial structure and relaxing the lateral structure [21]. It was expected that 3 months following distraction, the medial and lateral soft tissues would recover to some degree, and the TT correction would be maintained in some cases [1]. Based on this line of thinking, a fixed medial ankle joint distraction arthroplasty was used to further open the medial joint space and correct the TT angle in our later patients [34]. Tellisi et al. [35] reported 23 ankle joint distraction arthroplasty cases, and 6 of them were combined with SMOT to correct distal tibial deformities; however, no cases in this series focused on the increased TT angle. To date, no clinical study has reported the outcomes of SMOT combined with distraction arthroplasty for varus ankle osteoarthritis with increased TT angles.

**Fig. 3:**
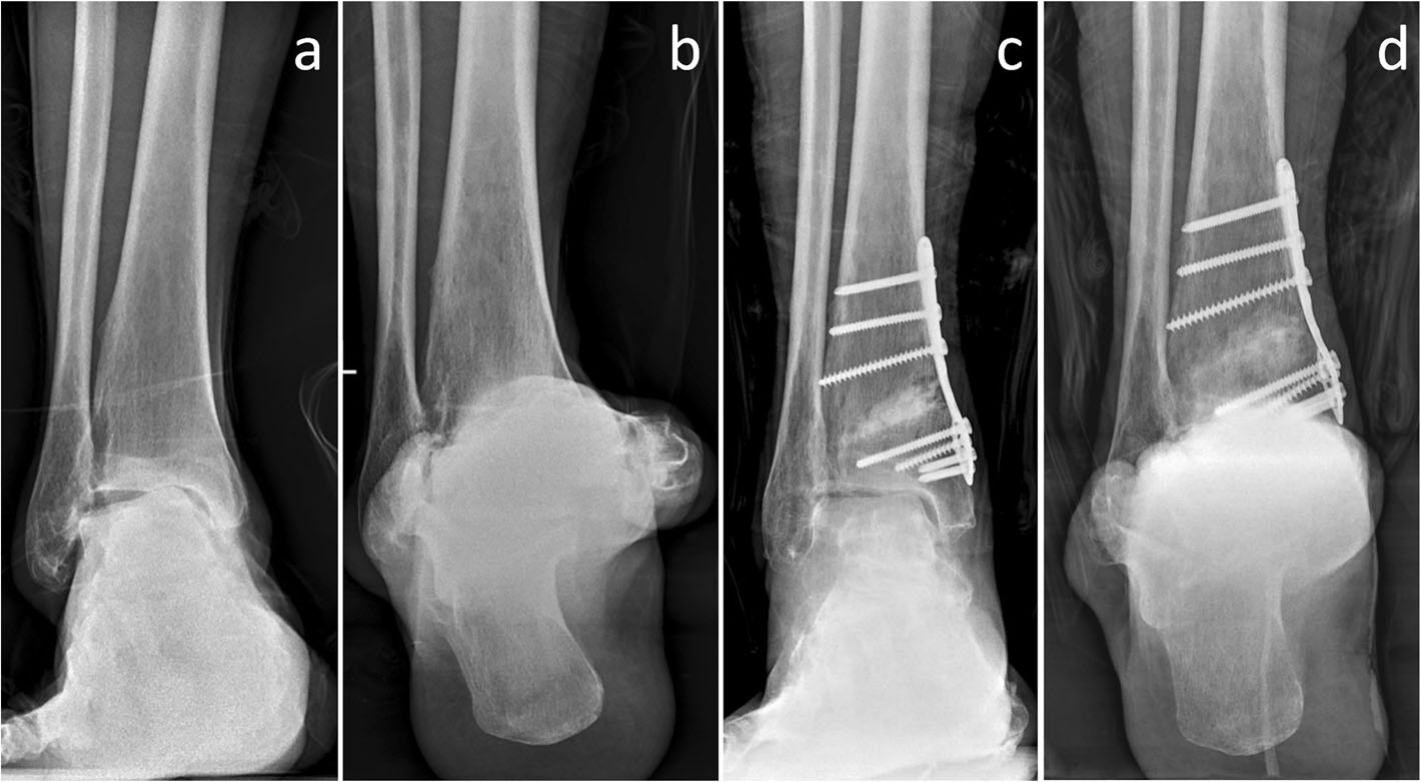
A 58-year-old male patient. The preoperative anterior-posterior view (**a**) and Saltzman view (**b**) showed stage 3a varus ankle osteoarthritis. The preoperative talar tilt angle was 14.3°. The patient was treated with supramalleolar osteotomy and modified Brostrom procedures. The 1-year postoperative scans showed that the alignment was better than that of the preoperative conditions, the functional outcomes were improved, and the modified Takakura stage was improved to stage 2 (**c**, **d**), but the talar tilt angle was still larger than normal (6.4°)

**Fig. 4:**
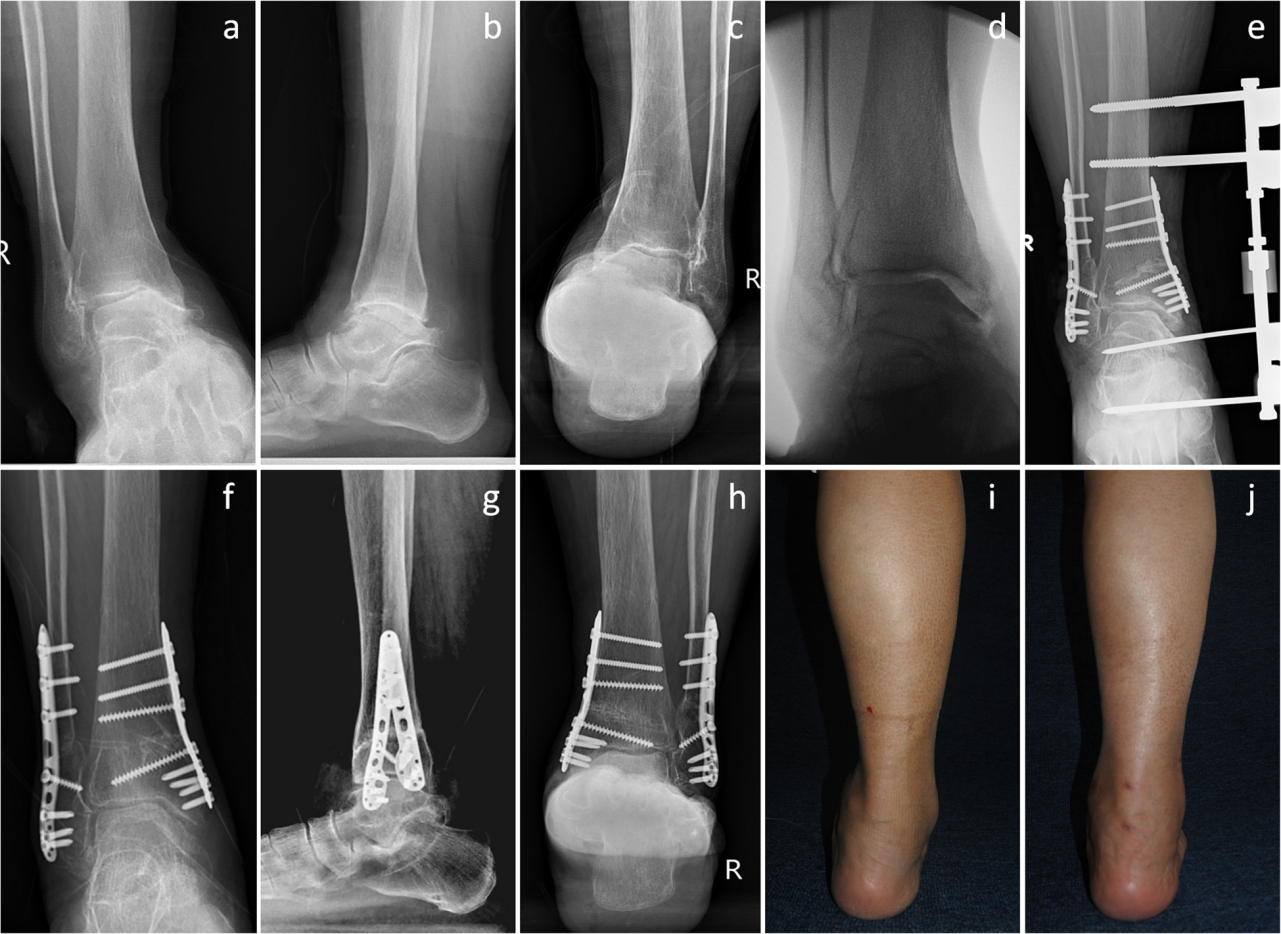
A 64-year-old female patient. The preoperative anterior-posterior view (**a**), lateral view (**b**), and Saltzman view (**c**) showed stage 3a varus ankle osteoarthritis. The preoperative talar tilt angle was 13.2°. The valgus stress view showed that the medial malleolus joint space could be opened, and the talar tilt could be corrected (**d**). The patient was treated with supramalleolar osteotomy, fibular osteotomy, and medial distraction arthroplasty (**e**). The 1-year postoperative scans showed that the normal alignment was maintained; the talar tilt angle was decreased to 1.4°, and the modified Takakura stage was improved to stage 1 (**f**, **g**, **h**). The preoperative hindfoot showed severe varus deformity (**i**), and the alignment was corrected to normal angles postoperatively (**j**)

To the best of our knowledge, this is the first study to report the functional and radiological outcomes of combining SMOT and distraction arthroplasty to correct varus ankle osteoarthritis with large varus TT angles and is the first study to compare the results of this technique with SMOT. According to the current results, SMOT alone has a high rate of failed correction of the TT angle (31.3%, Fig. 3). However, in the SMOT with MDA group, the increased TT angles in all of the patients were corrected to normal angles, and the correction was maintained (Fig. 4). The mean postoperative TT angle in the SMOT with MDA group was significantly smaller than in the SMOT group.

The limitations of the current study include its retrospective design and the lack of information on the intraarticular changes. Additionally, we used more autografts in the SMOT group and more allografts in the SMOT with MDA group. This difference is because we used more allografts in our later patents to decrease the relative donor site complications of the autografts, and we did not find delayed unions or nonunions with the use of allografts. The follow-up time in the SMOT with MDA group was significantly shorter than that of the SMOT group, which may be because we just started to use this technique (SMOT with MDA) 5 years previously. Other limitations included a relatively small sample size and a large age range in the included patients, which may result in heterogeneity. Although the outcomes will change over time, our early results confirmed that the functional outcome of the SMOT procedure is good in terms of alleviating pain relief, correcting the malalignment, and reducing the signs of varus ankle osteoarthritis patients with increased TT angles. In addition, the combined use of SMOT and distraction arthroplasty could better correct the TT angle.

## Conclusions

In conclusion, SMOT combined with MDA can improve the clinical and radiological outcomes of varus ankle osteoarthritis with large TT angles. This procedure may be helpful to restore the weight-bearing alignment of the ankle joint and to correct the TT angle that opens the medial ankle joint space. However, well-designed prospective comparative studies are still needed to further confirm the outcomes of medial distraction for increased TT angles.

## Abbreviations

AOFAS: American Orthopaedic Foot and Ankle Society
AOS: Ankle Osteoarthritis Scale
HFA: Hindfoot alignment angle
MDA: Medial distraction arthroplasty
ROM: Range of motion
SMOT: Supramalleolar osteotomy
TAS: Tibial articular surface angle
TC: Tibiocrural angle
TLS: Tibial lateral surface angle
TMM: Tibial medial malleolar angle
TT: Talar tilt angle
